# Bile acid receptor TGR5, NADPH Oxidase NOX5-S and CREB Mediate Bile Acid-Induced DNA Damage In Barrett’s Esophageal Adenocarcinoma Cells

**DOI:** 10.1038/srep31538

**Published:** 2016-08-11

**Authors:** Dan Li, Weibiao Cao

**Affiliations:** 1 Department of Medicine, Rhode Island Hospital and Warren Alpert Medical School of Brown University, Providence, RI, USA; 2 Department of Pathology, Rhode Island Hospital and Warren Alpert Medical School of Brown University, Providence, RI, USA.

## Abstract

The mechanisms whereby bile acid reflux may accelerate the progression from Barrett’s esophagus (BE) to esophageal adenocarcinoma (EA) are not fully understood. In this study we found that bile acid taurodeoxycholic acid (TDCA) significantly increased the tail moment (TM) and histone H2AX phosphorylation in FLO-1 EA cells, an increase which was significantly decreased by knockdown of TGR5. Overexpression of TGR5 significantly increased TDCA-induced TM increase and H2AX phosphorylation. In addition, NADPH oxidase inhibitor diphenylene iodonium significantly inhibited the TDCA-induced increase in TM and H2AX phosphorylation. TDCA-induced increase in TM and H2AX phosphorylation was significantly decreased by knockdown of NOX5-S and overexpression of NOX5-S significantly increased TDCA-induced increase in the tail moment and H2AX phosphorylation. Furthermore, TDCA significantly increased cAMP response element binding protein (CREB) phosphorylation in FLO-1 cells. Knockdown of CREB significantly decreased TDCA-induced increase in NOX5-S mRNA and the tail moment. Conversely, overexpression of CREB significantly increased TDCA-induced TM increase. We conclude that TDCA-induced DNA damage may depend on the activation of TGR5, CREB and NOX5-S. It is possible that in Barrett’s patients bile acids may activate NOX5-S and increase reactive oxygen species (ROS) production via activation of TGR5 and CREB. NOX5-S-derived ROS may cause DNA damage, thereby contributing to the progression from BE to EA.

The major risk factor for esophageal adenocarcinoma (EA) is gastroesophageal reflux disease (GERD) complicated by Barrett’s esophagus (BE). The mechanisms of the progression from BE to EA are not fully understood. Bile acids may contribute to the progression from BE to EA^1^,^2^ since (a) animals with surgical diversion of duodenal contents into the lower esophagus develops EA^3^,^4^,^5^; (b) In an *in vitro* experiment immortalized non-transformed esophageal Barrett cells become tumorigenic after repetitive exposure to bile salts in an acid environment over 65 weeks^6^. However, mechanisms whereby bile acids promote the development of EA are not known.

Bile acids have been reported to cause DNA damage^7^, among which double-strand breaks (DSBs) are the most harmful form. Persistent DSBs may cause chromosomal abnormalities including translocations and deletions^8^ and induce genomic instability, thus contributing to the tumorigenesis. How bile acids cause DNA damage is not clear. Bile acid deoxycholic acid (DCA)-induced DNA damage has been reported to be partially dependent on inducible nitric oxide synthase (iNOS) and nitric oxide (NO)^9^ and to be mediated by reactive oxygen species (ROS) since pretreatment with N-acetyl-l-cysteine (a ROS scavenger) prevented DNA damage induced by DCA^10^. We have previously shown that the bile acid-induced H_2_O_2_ production is mediated by activation of NADPH oxidase (NOX) NOX5-S and a bile acid receptor TGR5 in EA FLO-1 cells^11^. TGR5 is a G-protein-coupled receptor and plays an important role in bile acid-regulated lipid metabolism, energy homeostasis, and glucose metabolism^12^,^13^,^14^. Therefore, we examined the role of TGR5 and NOX5-S in bile acid-induced DNA damage. We find that the bile acid taurodeoxycholic acid-induced DNA damage is mediated by the activation of TGR5, NADPH oxidase 5-S (NOX5-S) and the cyclic AMP-response element-binding protein (CREB).

## Results

### Taurodeoxycholic acid (TDCA)-induced DNA damage in FLO-1 EA cells

**Figure 1:**
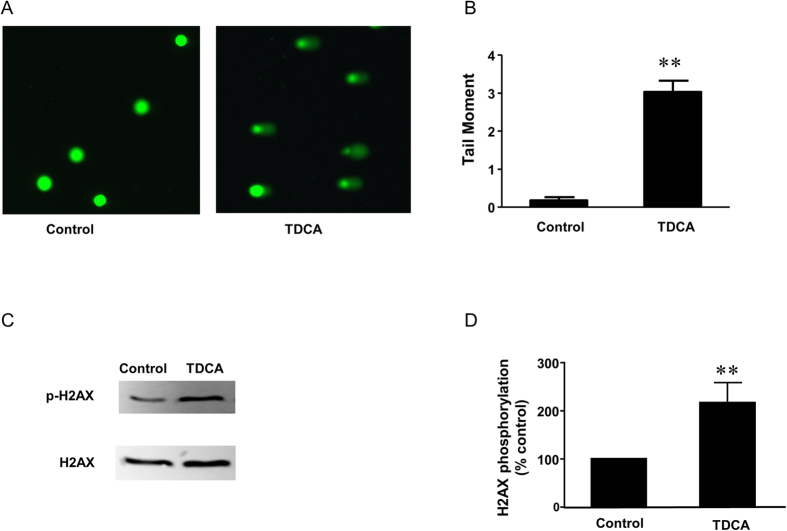
Bile acid taurodeoxycholic acid (TDCA) causes DNA damage. (**A**) Typical images and (**B**) summarized data showed that TDCA treatment (10^−11^ M, 24 hours) significantly increased tail moment, suggesting that TDCA may cause DNA damage in FLO-1 EA cells. Note that the presence of comet “tails” in the images indicates DSBs in FLO-1 cells. N = 162 cells of 3 experiments (control) and 208 cells of 3 experiments (TDCA group). (**C**) Typical images and (**D**) summarized data showed that TDCA treatment (10^−11^ M, 24 hours) significantly increased H2AX phosphorylation (N = 9), suggesting that TDCA may cause double stranded DNA break in FLO-1 EA cells. t test, **P < 0.0001.

To investigate whether TDCA causes DNA damage, FLO-1 cells, a human Barrett’s adenocarcinoma cell line derived from human Barrett’s esophageal adenocarcinoma, were incubated with 10^−11^M TDCA for 24 hours. DNA damage was examined by a Comet Assay. This assay is based on the ability of denatured cleaved DNA fragments to migrate out of the cell under the influence of an electric potential, whereas undamaged supercoiled DNA remains within the confines of the cell membrane. The DNA damage is quantitated by measuring the tail moment, which is defined as the product of the tail length and the fraction of total DNA in the tail (tail moment = tail length x % of DNA in the tail). Figure 1 showed that TDCA treatment significantly increased tail moment from 0.2 ± 0.08 to 3.1 ± 0.3 (t test, P ＜ 0.0001), suggesting that TDCA may cause DNA damage in FLO-1 EA cells. To further confirm this result, we examined histone H2AX phosphorylation, which has been shown to be a marker of double stranded DNA break^15^,^16^. We found that TDCA significantly increased H2AX phosphorylation in FLO-1 cells (Fig. 1C,D), indicating that TDCA may cause double stranded DNA break.

### Role of TGR5 in TDCA-induced DNA damage

**Figure 2:**
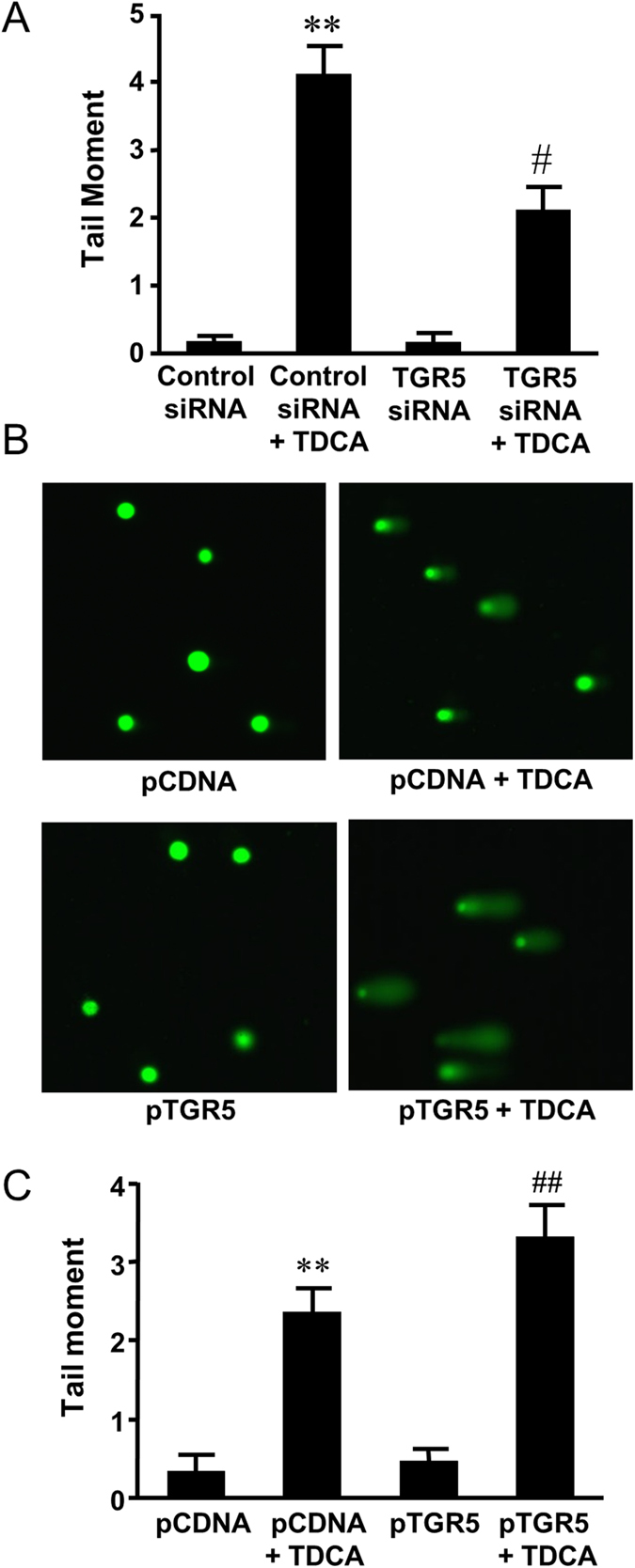
The role of TGR5 in TDCA-induced DNA damage. (**A**) Bile acid TDCA significantly increased the tail moment. Knockdown of TGR5 significantly decreased the tail moment in response to TDCA treatment in FLO-1 cells (N = 134-253 cells of 3 experiments). (**B**) Representative images of the Comet Assay at 4X magnification in FLO-1 cells transfected with pcDNA3.1 or TGR5 plasmid with or without TDCA treatment (10^−11^ M, 24 hours). C) Overexpression of TGR5 significantly increased the tail moment in response to TDCA treatment in FLO-1 cells (N = 190–263 cells of 3 experiments). These data suggest that TGR5 may mediate bile acid-induced DNA damage. ANOVA **P < 0.0001, compared with Control siRNA group or pCDNA group; ^#^P < 0.0001, compared with Control siRNA group plus TDCA group; ^##^P < 0.05, compared with Control pCDNA group plus TDCA group.

**Figure 3:**
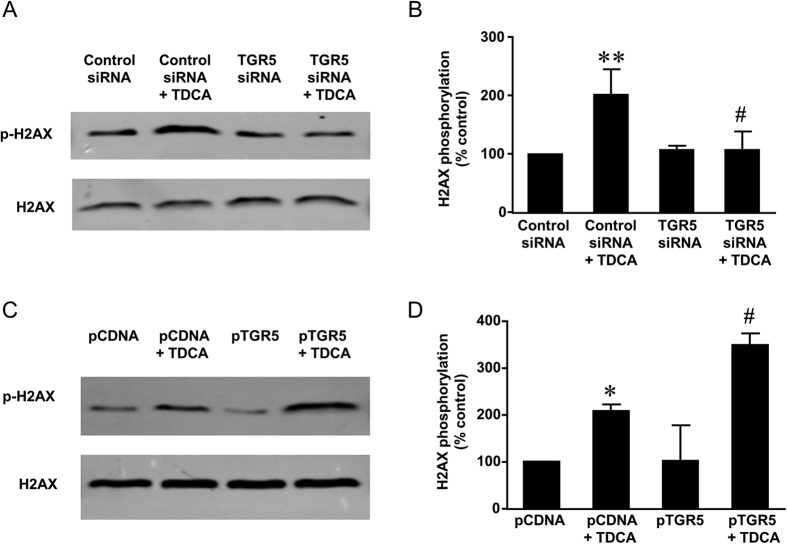
The role of TGR5 in TDCA-induced H2AX phosphorylation. (**A**) Typical images of Western blot analysis and (**B**) summarized data showed that TDCA (10^−11^ M, 24 hours) significantly increased H2AX phosphorylation, an increase which was significantly decreased by knockdown of TGR5 (N = 3). (**C**) Typical images of Western blot analysis and (**D**) summarized data showed that overexpression of TGR5 enhanced TDCA-induced increase in H2AX phosphorylation in FLO-1 cells (N = 3). These data suggest that TGR5 may mediate TDCA-induced H2AX phosphorylation. ANOVA, *P < 0.05, **P < 0.01, compared with Control siRNA group or pCDNA group; ^#^P < 0.05, compared with Control siRNA plus TDCA group or pCDNA plus TDCA group.

We have previously shown that TGR5 mediates bile acid-induced increase in NOX5-S expression. Therefore, we examined the role of TGR5 in TDCA-induced DNA damage. We used TGR5 siRNA, which had been shown by us to effectively knock down TGR5^11^, to knock down TGR5. Figure 2A showed that knockdown of TGR5 significantly decreased TDCA-induced increase in tail moment from 4.1 ± 0.4 to 2.1 ± 0.3 (ANOVA, P ＜ 0.0001) in FLO-1 cells. In addition, overexpression of TGR5 by transfection of FLO-1 cells with TGR5 plasmid significantly increased TDCA-induced increase in tail moment from 2.4 ± 0.3 to 3.3 ± 0.4 (ANOVA, P ＜ 0.05, Fig. 2B,C). Similarly, knockdown of TGR5 significantly decreased TDCA-induced increase in H2AX phosphorylation (Fig. 3A,B) and overexpression of TGR5 significantly enhanced TDCA-induced H2AX phosphorylation (Fig. 3C,D). These data suggest that TGR5 may mediate TDCA-induced DNA damage.

### Role of NOX5-S in acid-induced DNA damage

**Figure 4:**
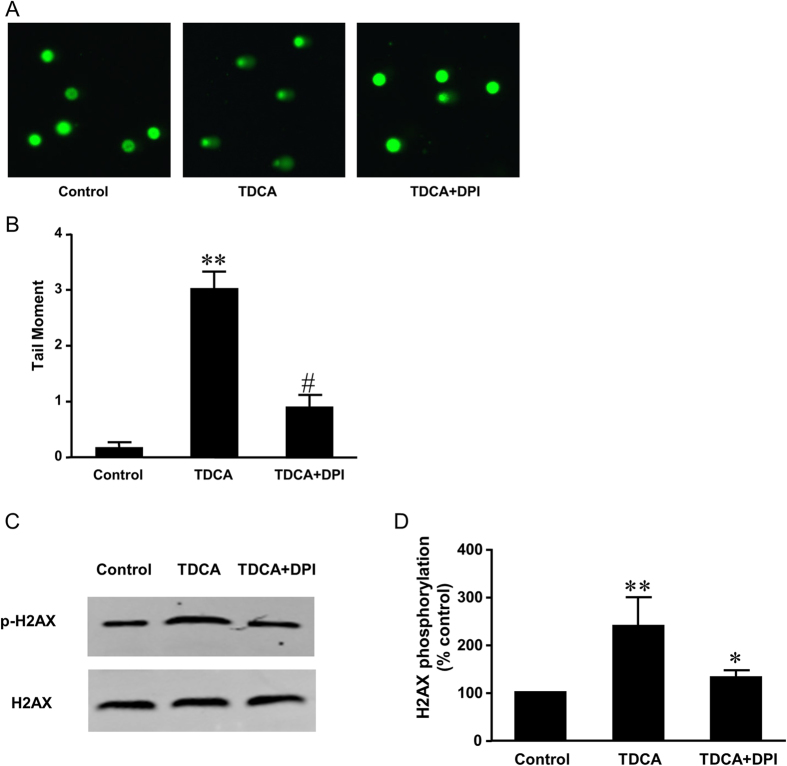
The role of NADPH oxidases in TDCA induced DNA damage. (**A**) Representative images of the Comet Assay at 4X magnification in FLO-1 cells treated with or without TDCA treatment (10^−11^ M,24 hours) in the presence or absence of NADPH oxidase inhibitor diphenyleneiodonium (DPI 10^−5^ M). (**B**) TDCA-induced increase in tail moment was significantly reduced by DPI (N = 149–208 cells of 3 experiments). (**C**) Typical images of Western blot analysis and (**D**) summarized data showed that TDCA (10^−11^ M, 24 hours) significantly increased H2AX phosphorylation, an increase which was significantly decreased by DPI (N = 3). These data suggest that NADPH oxidases may be involved in TDCA-induced DNA damage. ANOVA **P < 0.0001, compared with control group; ^#^P < 0.0001, *P < 0.05, compared with TDCA group.

We have shown that TDCA increases NOX5-S expression and H_2_O_2_ production in FLO-1 cells and a Barrett’s cell line BAR-T^11^. Thus we examined the role of NOX5-S in TDCA induced DNA damage. Firstly we used NADPH oxidase (NOX) inhibitor diphenyleneiodonium (DPI)^17^. We found that TDCA-induced increase in tail moment was significantly reduced by DPI from 3.0 ± 0.3 to 1.0 ± 0.2 (ANOVA, P ＜ 0.0001, Fig. 4A,B). In addition, DPI significantly inhibited TDCA-induced increase in H2AX phosphorylation (Fig. 4C,D). These data suggest that NADPH oxidases may be involved in TDCA-induced DNA damage.

**Figure 5:**
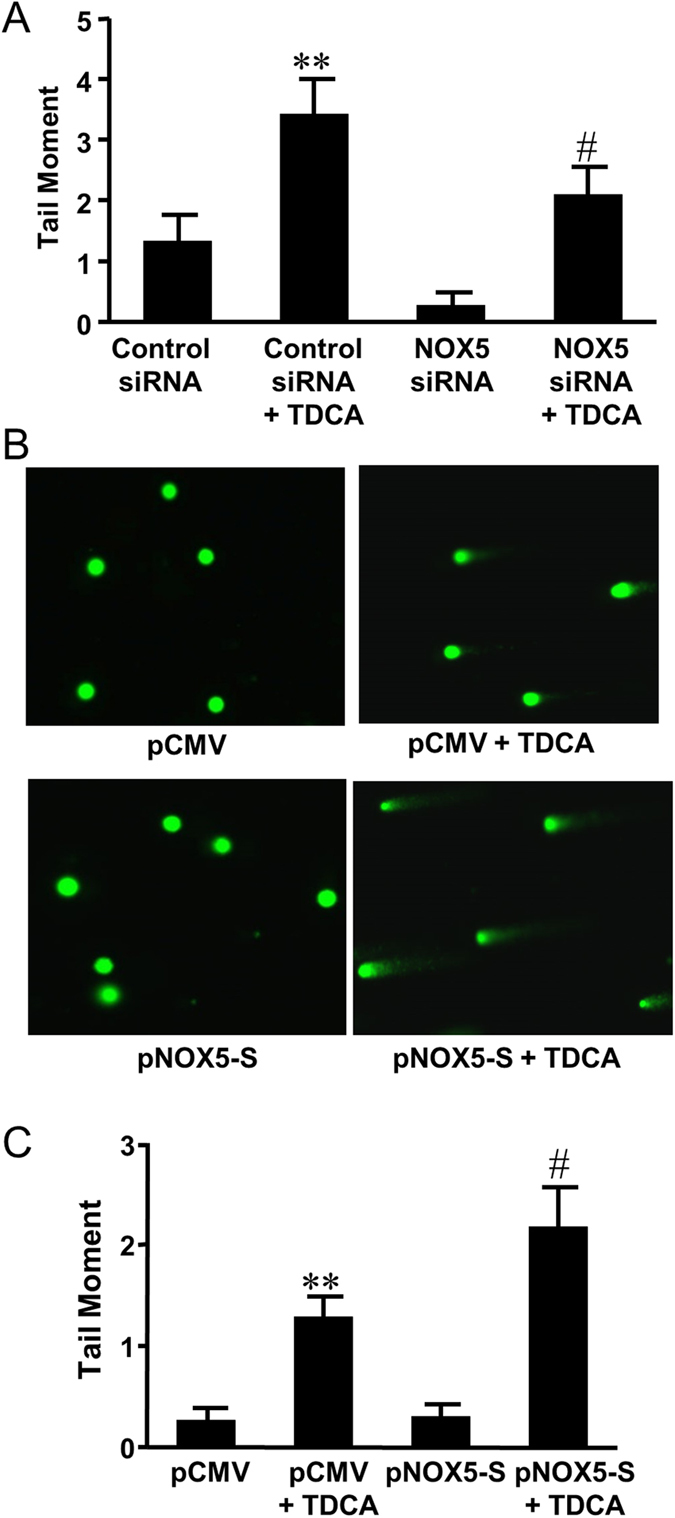
The role of NOX5-S in TDCA-induced DNA damage. (**A**) Knockdown of NOX5-S significantly decreased the tail moment in response to TDCA treatment in FLO-1 cells (N = 130–171 cells of 3 experiments). (**B**) Representative images of the Comet Assay at 4X magnification in FLO-1 cells transfected with pCMV or NOX5-S plasmid with or without TDCA treatment (10^−11^ M, 24 hours). (**C**) Overexpression of NOX5-S significantly increased the tail moment in response to TDCA treatment in FLO-1 cells (N = 136–172 cells of 3 experiments). These data suggest that NOX5-S may mediate TDCA-induced DNA damage in FLO-1 cells. ANOVA **P < 0.01, compared with Control siRNA group or pCMV group; ^#^P < 0.05, compared with Control siRNA group plus TDCA group or pCMV plus TDCA group.

**Figure 6:**
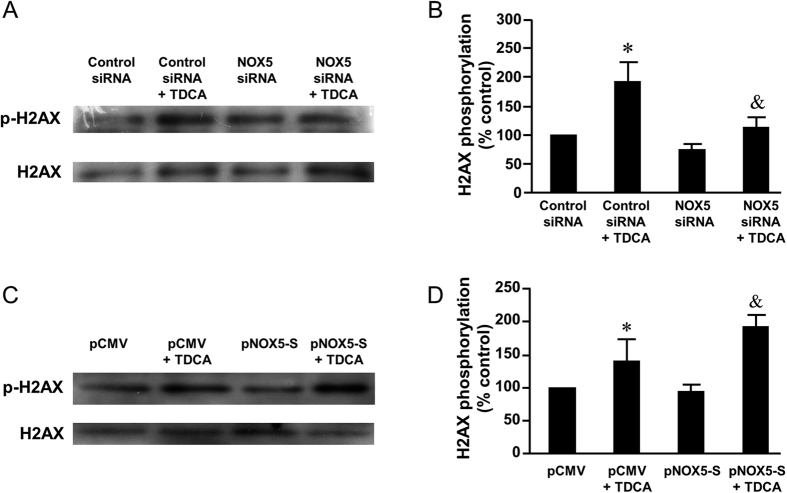
The role of NOX5-S in TDCA-induced H2AX phosphorylation. (**A**) Typical images of Western blot analysis and (**B**) summarized data showed that TDCA significantly increased H2AX phosphorylation, an increase which was significantly decreased by knockdown of NOX5-S (N = 3). (**C**) Typical images of Western blot analysis and (**D**) summarized data showed that overexpression of NOX5-S enhanced TDCA-induced increase in H2AX phosphorylation in FLO-1 cells (N = 3). These data suggest that NOX5-S may mediate TDCA-induced H2AX phosphorylation. ANOVA, *P < 0.05, compared with Control siRNA group or pCMV group; ^&^P < 0.05, compared with Control siRNA plus TDCA group or pCMV plus TDCA group.

Then we used NOX5 siRNA, which had been shown by us to effectively knock down NOX5-S^18^, to knock down NOX5-S. Figure 5A showed that knockdown of NOX5-S significantly decreased TDCA induced increase in tail moment from 3.4 ± 0.6 to 2.1 ± 0.4 (ANOVA, P ＜ 0.05) in FLO-1 cells (Fig. 5A), suggesting that NOX5-S may be involved in TDCA-induced DNA damage. Furthermore, TDCA significantly increased H2AX phosphorylation in FLO-1 cells, an increase which was significantly reduced by knockdown of NOX5-S (Fig. 6A,B). In addition, overexpression of NOX5-S by transfection with NOX5-S plasmid significantly increased TDCA induced increase in tail moment from 1.3 ± 0.2 to 2.1 ± 0.4 (ANOVA, P ＜ 0.05, Fig. 5B,C) in FLO-1 cells. Similarly, overexpression of NOX5-S significantly increased histone H2AX phosphorylation in response to TDCA treatment in FLO-1 cells (Fig. 6C,D). The data suggest that NOX5-S may contribute to TDCA-induced DNA damage in FLO-1 cells.

### Role of CREB in TDCA-induced DNA damage

**Figure 7:**
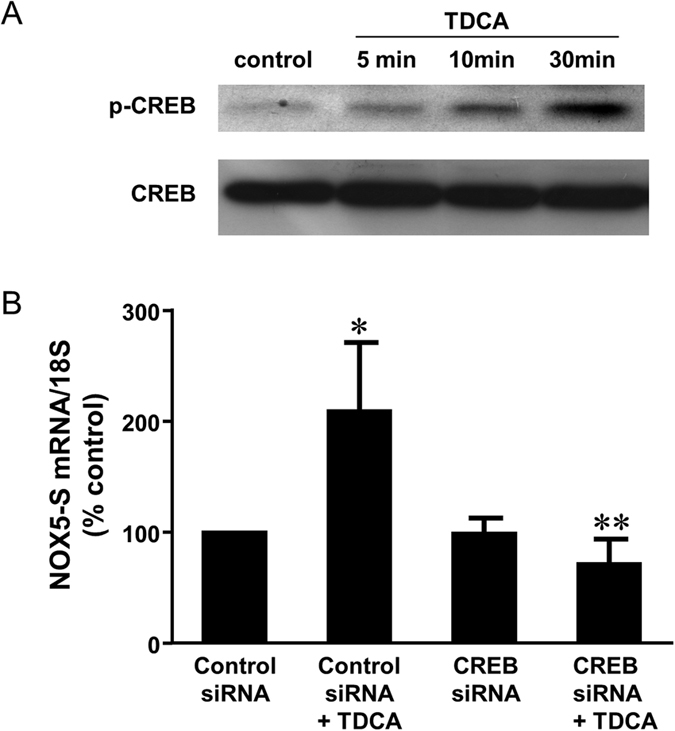
The role of CREB in TDCA-induced NOX5-S expression. (**A**) TDCA treatment (10^−11^ M) significantly increased phosphorylation of CREB in a time-dependent manner in FLO-1 cells, indicating that TDCA may activate CREB. B) TDCA significantly increased NOX5-S mRNA levels, an increase which was significantly decreased by knockdown of CREB, suggesting that CREB may mediate TDCA-induced increase in NOX5-S expression. N = 4, ANOVA *P < 0.05, compared with Control siRNA group; **P < 0.02, compared with Control siRNA plus TDCA group.

We have reported that cyclic AMP response element binding protein (CREB) is responsible for acid-induced expression of NOX5-S in SEG1 cells (a possible lung carcinoma cell line)^19^. However, whether CREB mediates bile acid-induced NOX5-S expression and DNA damage is not known. We found that TDCA treatment significantly increased phosphorylation of CREB in a time-dependent manner in FLO-1 cells (Fig. 7A), indicating that TDCA may activate CREB.

**Figure 8:**
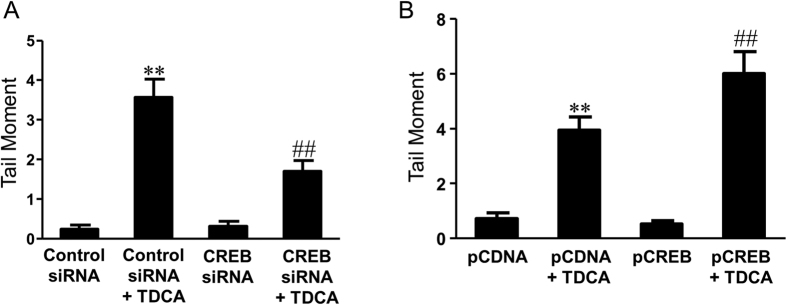
The role of CREB in TDCA-induced DNA damage. (**A**) Knockdown of CREB significantly decreased the tail moment in response to TDCA treatment in FLO-1 cells (N = 187–308 cells of 3 experiments). (**B**) Overexpression of CREB significantly increased the tail moment in response to TDCA treatment in FLO-1 cells (N = 171–369 cells of 3 experiments). These data suggest that CREB may mediate TDCA-induced DNA damage in FLO-1 cells. ANOVA **P < 0.0001, compared with Control siRNA group or pCDNA group; ^##^P < 0.001, compared with Control siRNA group plus TDCA group or pCDNA plus TDCA group.

Next we examine the role of CREB in TDCA-induced NOX5-S expression and DNA damage. We used CREB siRNA, which had been shown by us to effectively knock down CREB^18^, to knock down CREB. Knockdown of CREB significantly decreased TDCA-induced increase in NOX5-S mRNA levels from 209% to 72% control (Fig. 7B), suggesting that CREB may mediate TDCA-induced increase in NOX5-S expression. In addition, knockdown of CREB significantly decreased TDCA-induced increase in tail moment from 3.6 ± 0.5 to 1.7 ± 0.3 (ANOVA, P ＜ 0.001, Fig. 8A) in FLO-1 cells. Furthermore, overexpression of CREB by transfection with CREB plasmid significantly increased TDCA-induced increase in tail moment from 3.9 ± 0.2 to 5.9 ± 0.8 (ANOVA, P ＜ 0.001, Fig. 8B) in FLO-1 cells. The data suggest that CREB may be involved in TDCA-induced DNA damage in FLO-1 cells.

## Discussion

There is increasing evidence that bile acids may contribute to the progression from BE to EA^1^,^2^. Bile acids have been shown to cause DNA damage^7^, which is mediated by reactive oxygen species (ROS)^10^. However, the mechanisms of bile acid-induced DNA damage are not fully understood.

We have previously shown that the NADPH oxidase NOX5-S is present in EA FLO-1 cells and mediates the bile acid-induced increase in H_2_O_2_ production^11^. In addition, the bile acid-induced increase in NOX5-S expression may be mediated by activation of the TGR5 receptor and Gαq protein^11^. Therefore, we hypothesized that bile acid-induced H_2_O_2_ production via activation of NOX5-S and TGR5 may contribute to the DNA damage.

We firstly examined the role of bile acid receptor TGR5 in bile acid-induced DNA damage. We found that TGR5 may mediate bile acid-induced DNA damage since 1) knockdown of TGR5 significantly decreased TDCA-induced increase in tail moment and H2AX phosphorylation in FLO-1 cells; 2) overexpression of TGR5 significantly increased TDCA-induced increase in tail moment and H2AX phosphorylation.

Next we examined the role of NOX5-S in bile acid-induced DNA damage since we have shown that the NADPH oxidase isoform NOX5-S is present in FLO-1 EA cells and may be a source of reactive oxygen species^11^. In addition, bile acid increases NOX5-S expression via activation of the TGR5 receptor and Gαq protein^11^. We found that NOX5-S mediates TDCA-induced DNA damage because 1) TDCA-induced increase in tail moment was significantly reduced by NADPH oxidase inhibitor DPI; 2) knockdown of NOX5-S significantly decreased TDCA induced increase in tail moment in FLO-1 cells; 3) overexpression of NOX5-S by transfection with NOX5-S plasmid significantly increased TDCA induced increase in tail moment. To further confirm this result, DNA damage was examined by measurement of histone H2AX phosphorylation, which has been shown to be a marker of double stranded DNA break^15^,^16^. We found that TDCA significantly increased H2AX phosphorylation in FLO-1 cells, an increase which was significantly reduced by DPI and knockdown of NOX5-S. In addition, overexpression of NOX5-S significantly increased histone H2AX phosphorylation in response to TDCA treatment in FLO-1 cells, further confirming our result that NOX5-S may contribute to TDCA-induced DNA damage in FLO-1 cells.

In addition, we examined the role of cyclic AMP-response element-binding protein (CREB, a transcription factor) in TDCA-induced DNA damage since we have found that CREB is responsible for acid-induced expression of NOX5-S. CREB is a Ca^2+^-dependent and ubiquitous transcription factor and binds the consensus CRE DNA sequence TGACGTCA (27, 51). The ability of CREB to activate transcription requires its phosphorylation on serine 133^20^. CREB is a 43 kD basic leucine‐zipper transcription factor that regulates gene expression through the cAMP‐dependent or independent signal transduction pathways^21^,^22^. We have previous identified two CRE binding elements TGACGAGA and TGACGCTG in the NOX5-S gene promoter^18^, confirming the role of CREB in the regulation of NOX5-S expression. However, whether bile acid activates CREB is not known. We found that TDCA significantly increased CREB phosphorylation in a time-dependent manner, indicating that bile acid may activate CREB. We also found that CREB may mediate bile acid-induced increase in NOX5-S expression because knockdown of CREB significantly decreased TDCA-induced increase in NOX5-S mRNA levels. In addition, knockdown of CREB significantly decreased TDCA-induced increase in tail moment in FLO-1 cells and overexpression of CREB significantly increased TDCA-induced increase in tail moment. These data suggest that CREB may be involved in TDCA-induced NOX5-S expression and DNA damage in FLO-1 cells.

In conclusion, bile acid causes DNA damage via activation of TGR5, CREB and NOX5-S in FLO-1 cells. Although these data were obtained in an *in vitro* cell line, which is a limitation of this study, our data imply that in Barrett’s esophagus bile acids present in the refluxate activate TGR5 and CREB, thereby upregulating NOX5-S. High levels of ROS derived from NOX5-S may cause DNA damage, thereby contributing to the progression from BE to EA.

## Materials and Methods

### Cell Culture and Treatment

The human Barrett’s adenocarcinoma cell line FLO-1^23^ was obtained from Dr. David Beer (University of Michigan). The FLO-1 cells were cultured in Dulbecco’s modified Eagle’s medium (DMEM) containing 10% fetal bovine serum and antibiotics.

For taurodeoxycholic acid (TDCA) treatment, FLO-1 cells were incubated with 10^−11^ M TDCA for 24 h. For diphenyleneiodonium (DPI) treatment, FLO-1 cells were pretreated with DPI (10^−5^ M), or culture medium (control) for 1 h and then treated with or without TDCA (10^−11^ M) for an additional 24 h. Finally, the culture medium and cells were collected for measurements.

### Small interfering RNA (siRNA) and plasmid transfection

The protocol for small interfering RNA (siRNA) transfection has been described by us in our previous publication^24^. 60 pmol of siRNA duplex of NOX5, TGR5, CREB or control siRNAs formulated into liposomes were added to each well.

The protocol for plasmid transfection was similar to that in our previous publication^24^. 0.5 μg of plasmids (pCMV-NOX5-S, pCMV, pCDNA3.1-TGR5, pCDNA3.1, RSV-CREB or RSV) formulated into liposomes were added to each well. The pCMV-tag5a-NOX5- S plasmid was obtained from Dr. David Lambeth (Emory University School of Medicine, Atlanta, GA); CREB expression vector was generously provided to us by Dr. Marc R. Montminy (The Salk Institute for Biological Studies, San Diego, CA)^20^.

Twenty-four hours after transfection with siRNA or plasmid, cells were treated with or without TDCA 10^−11^ M in culture medium (pH 7.2; without phenol red) for 24 h, and then the culture medium and cells were collected for measurements.

### Reverse transcription-PCR

Total RNA was extracted by Trizol reagent (Thermo Fisher Scientific, Waltham, MA) and 1.5 μg of total RNAs were reversely transcribed by using a SuperScript First-Strand Synthesis System for reverse transcription-PCR (Thermo Fisher Scientific, Waltham, MA).

### Quantitative real-time PCR

Real-time PCR was performed as we previously described^11^,^24^. The primers used were as follows: NOX5-S sense (5**′**-AAGACTCCATCACGGGGCTGCA-3**′**), NOX5-S antisense (5**′**-CCTTCAGCACCTTGGCCAGA-3**′**), 18S sense (5**′**-CGGACAGGATTGACAGATTGATAGC-3**′**), and 18S antisense (5**′**-TGCCAGAGTCTCGTTCGTTATCG-3**′**). Reactions were carried out in an Applied Biosystems StepOnePlus real-time PCR system for one cycle at 94 °C for 5 min; 40 cycles at 94 °C for 30 s, 59 °C for 30 s, and 72 °C for 30 s; 1 cycle at 94 °C for 1 min; and 1 cycle at 55 °C for 30 s.

### Western Blot Analysis

Cells were lysed and Western blot analysis were carried out as we previously described 18,25. The primary antibodies used were phospho-histone H2AX (Ser139) antibody (1:1000, Cell signaling technology, cat # 2577), H_2_AX antibody (1:1000, Cell signaling technology, cat # 2595), phospho-CREB (Ser133) antibody (1:1000, Cell signaling technology, cat # 9191) and CREB antibody (1:1000, Cell signaling technology, cat # 9197).

### Comet Assay

The comet assay was done as we previously reported^24^. For each sample, 100 randomly selected cells (50 cells from each of the two duplicate slides) were analyzed. The tail moment was analyzed using TriTek CometScore TM software.

### Materials

Human TGR5 and CREB siRNA were purchased from Santa Cruz biotechnology Inc., Dallas, Texas; human NOX5 siRNA from Ambion Inc. (Austin, TX), and CREB siRNA from Upstate (Charlottesville, VA ). DPI, Triton X- 100, phenylmethylsulfonyl fluoride, DMEM, antibiotics, and other reagents were purchased from Sigma-Aldrich.

### Statistical Analysis

Data is expressed as mean ± S.E. Statistical differences between two groups were determined by Student’s *t* test. Differences among multiple groups were tested using analysis of variance (ANOVA) and checked for significance using Fisher’s protected least significant difference test.

## Additional Information

**How to cite this article**: Li, D. and Cao, W. Bile acid receptor TGR5, NADPH Oxidase NOX5-S and CREB Mediate Bile Acid-Induced DNA Damage In Barrett’s Esophageal Adenocarcinoma Cells. *Sci. Rep.* **6**, 31538; doi: 10.1038/srep31538 (2016).
